# Bioprospecting endophytic fungi of forest plants for bioactive metabolites with anticancer potentials

**DOI:** 10.1038/s41598-025-10372-9

**Published:** 2025-07-21

**Authors:** El-Sayed R. El-Sayed, Magdalena Kloc, Julita Kulbacka, Anna Choromańska, Monika Bielecka, Tomasz Strzała, Jacek Łyczko, Filip Boratyński

**Affiliations:** 1 Department of Food Chemistry and Biocatalysis, Wrocław University of Environmental and Life Sciences, Norwida 25, 50-375 Wrocław, Poland; 2 Plant Research Department, Nuclear Research Center, Egyptian Atomic Energy Authority, Cairo, Egypt; 3 Faculty of Medicine, Wrocław Medical University, Wrocław, Poland; 4 “Scientific Students” Group No. 148 of the Department of Molecular and Cellular Biology, Wroclaw Medical University, Wrocław, Poland; 5 Department of Molecular and Cellular Biology, Faculty of Pharmacy, Wroclaw Medical University, Borowska 211A, 50-556 Wroclaw, Poland; 6 Department of Immunology and Bioelectrochemistry, State Research Institute Centre for Innovative Medicine, Santariškiu˛ G. 5, 08406 Vilnius, Lithuania; 7 Department of Pharmaceutical Biology and Biotechnology, Wroclaw Medical University, Borowska 211A, 50-556 Wroclaw, Poland; 8 Department of Genetics, Wrocław University of Environmental and Life Sciences, Kożuchowska 7, 51-631 Wrocław, Poland

**Keywords:** Anticancer, Bioactivities, Cytotoxic, Endophytes, Gamma irradiation, GC–MS, Microbiology, Applied microbiology, Fungi

## Abstract

Cancer continues to be a major cause of mortality worldwide, emphasizing the critical need for innovative and more effective anticancer drugs with enhanced efficacy and minimal side effects. In response, researchers have begun investigating the largely unexplored metabolites produced by fungal endophytes for the development of novel therapeutics. In this regard, the present work aims to assess the anticancer potential of the fungal endophytes of forest plants (local forest in Wrocław, Poland), which remains yet unexamined. Fungal endophytes were isolated from their host plants, purified, grown and their extracts were separately prepared from cell-free culture media and biomass. The prepared extracts were tested for their cytotoxic potentials against human melanoma, breast adenocarcinoma, lung carcinoma, and normal cell lines using the MTT-based assay. Cultures of these isolates were exposed to several doses of ^60^Co gamma rays to study their effects on the cytotoxic potential of their cell-based and extracellular extracts against the beforementioned cell lines. Preparative thin-layer chromatography fractionation followed by GC–MS were applied to identify compounds in the active fractions. Five fungi, namely *Penicillium yarmokense* JAR-L1 (leaves of *Sorbus aucuparia*), *Penicillium sp.* sp. BRZ-B (bark of *Betula pendula*), *Fusarium avenaceum* KLON-B (bark of *Acer platanoides*), *Penicillium charlesii* SOS-B1 (bark of *Pinus sylvestris*), and *Umbelopsis isabellina* COR-L2 (leaves of *Corylus avellana*) showed high toxicity with increased specificity for cancer cells rather than healthy cells. Exposure to gamma rays at specific doses enhanced the cytotoxic potentials of extracts of these fungi. Bioactive compounds in the active bands after prep-TLC were identified. Some of the identified compounds from active fractions such as squalene, fenchone, α-phellandrene, *p*-Cymene, α-thujone, and 18-norabietane are being reported for the first time from fungal cultures. The findings of this study indicate that endophytic fungi may represent a promising and untapped reservoir of bioactive compounds with cytotoxic effects which opens new avenues for scientific exploration and could contribute to the advancement of novel anticancer therapeutics.

## Introduction

Cancer comprises a group of diseases that can impact different organs, characterized by genetic mutations leading to uncontrolled cellular growth and their infiltration into healthy tissues^1^. These cancerous cells can also migrate to other areas of the body, forming secondary tumors. If this uncontrolled spread persists, it can ultimately result in death^2^. By 2050, the annual number of cancer-related deaths is projected to reach 17.5 million, driven by population growth, aging, and the impact of lifestyle and environmental factors^3^. Cancer continues to be a major cause of mortality worldwide, highlighting the urgent need for novel and more effective anticancer therapies^4^. Conventional treatments, such as chemotherapy and radiation, are often associated with severe side effects and the development of drug resistance, emphasizing the necessity for alternative therapeutic strategies^5^. Despite significant advancements in cancer treatment, the disease’s complexity and ability to adapt continue to present major challenges. Consequently, there is a pressing need for new drugs that offer improved efficacy with minimal adverse effects^6^.

Natural products have long been a cornerstone of drug discovery, providing structurally diverse compounds with significant pharmacological potential^7^. Exploring new biological sources, including plants, animals, and microorganisms, remains essential for identifying innovative therapeutic agents with enhanced anticancer properties^8^. Plant extracts due to their treatment effectiveness and low cytotoxicity have become beneficial in managing the malignant tendency of cancers^9^. For example, taxol is a chemotherapeutic diterpenoid produced by extraction process from the bark of the Pacific yew tree; however, these methods were inefficient and environmentally costly^7^. Furthermore, the yew trees are rare and grow very slowly, and a large amount of bark needs to be processed resulting in small yield^8^. Moreover, overharvesting these plants for producing anticancers affects the ecological balance^8^. Accordingly, exploring alternative non-plant sources for anticancer medications is crucial.

Endophytic fungi reside asymptomatically within plant tissues, engaging in symbiotic relationships with their hosts^10^. These microorganisms are prolific producers of secondary metabolites, many of which have demonstrated significant pharmacological activities^11^, including anticancer effects^12^. The unique environment within plant tissues allows endophytic fungi to biosynthesize novel compounds not found in other microorganisms^13^, making them valuable candidates for drug discovery^14^. Several different fungi can be isolated from one individual plant, creating unique microecosystems, unrepeatable even in a plant individual of the same species^15^. Also, endophytic fungi from slow-growing or rare and endangered plants can produce new bioactive compounds^16,17^. Notably, recent years have seen an unprecedented increase in discoveries related to endophytic fungi metabolites, underscoring their growing significance as valuable sources for novel, natural drug-like molecules^18^. For all of these reasons, we targeted endophytic fungi of forest plants to explore their cytotoxic potential. Additionally, the effect of gamma irradiation on the cytotoxic potentials of the promising fungi was also investigated. Generally, gamma radiation (at specific doses) can induce random mutagenesis yielding enhancement in the metabolic profile of cells^19^. Finally, chemical constituents of the active fractions were isolated and identified.

## Materials and methods

### Fungi, cultivation conditions, and preparation of extracts

Forty-three fungal strains were used to test the anticancer potential of their extracts^19,20^. The isolated fungi were deposited in the BioExplor culture collection of the Department of Food Chemistry and Biocatalysis, Wrocław University of Environmental and Life Sciences in under voucher number BioExplor2022.

Each fungal isolate was grown for 7 days at 30 °C and spore suspensions were collected and adjusted (10^5^ mL^−1^) using a hemocytometer. Spore suspensions were individually added under aseptic conditions to 250 mL Erlenmeyer flasks containing Sabouraud Dextrose Broth (50 mL, pH 6.0) medium. Flasks were incubated statically at 30 °C for 14 days.

Fungal cultures after completion of incubation were filtered through Whatman No.1 filter paper. The cell-free filtrate was extracted with equal volume of chloroform:methanol (9:1, v/v) under shaking overnight. Fresh biomass was ground using mortar and pestle until homogeneity and mixed with 25 mL mixture of chloroform:methanol (9:1, v/v) then sonicated for 1 h in an ultrasonic bath (20 kHz, 20 °C). Solvent layers were collected by a separating funnel, passed over sodium sulphate anhydrous, then evaporated under vacuum (at 40 °C). The resulting dry crude extract was dissolved in DMSO (1 mL) and used for testing.

### Cell culture

Three different cancer cell lines (ATCC, American Type Culture Collection) of human melanoma (A375), breast adenocarcinoma (MCF-7), and lung carcinoma (A549) and one normal cell line (Hfb-4) were used to evaluate the cytotoxic activities of the fungal extracts. Cells were cultured as a monolayer in Dulbecco’s modified Eagle’s medium supplemented with 10% Fetal Bovine Serum, and 1% of streptomycin/penicillin, and 1% l-glutamine. All chemicals were purchased from Sigma-Aldrich. The cells were maintained by incubation at 37 °C in a humidified atmosphere with 5% CO_2_ with two times sub-culturing in a week. All cells were tested for Mycoplasma contamination before experiments according to the European Pharmacopoeia (6th edition, 2007, section 2.6.7).

### Anticancer activity screening

Cytotoxicity of the prepared extracts was evaluated against the aforementioned cell lines using the MTT assay according to the method described by Van de Loosdrecht and co-workers^21^ with slight modifications. Cell lines at the count of 10^4^ cells/well in serum-free media were plated in a 96-well flat-bottom microplate (Falcon, NJ, USA). Wells were separately treated with 20 μL of the prepared extracts (either cell-free filtrate or biomass) for 24 h (in a humidified 5% CO_2_ atmosphere) at 37 °C. The MTT assay was conducted in presence of the extract. Meanwhile, wells (containing cells) treated with DMSO (0.1%, without fungal extract) served as controls. Taxol (50 µg mL^−1^) was used as the positive control. Then, MTT solution (10 μL, 5 mg mL^−1^) was added and wells were further incubated for 4 h till formation of purple color formazone crystals. The crystals were then dissolved in 100 μL of DMSO per well. Finally, the absorbances (570 nm) of the plates were recorded using a SunRise; TECAN, Inc., USA microplate reader after shaking for 5 s and the percentage of inhibition was expressed as Inhibition (%) = [100 − (A570 of treated cells/A570 of control cells) ] × 100.

### Fungal strains

Among all the collected and tested fungal endophytes five different fungi showed promising cytotoxic activities. These strains were *Penicillium yarmokense* JAR-L1, *Penicillium sp.* sp. BRZ-B, *Fusarium avenaceum* KLON-B, *Penicillium charlesii* SOS-B1, and *Umbelopsis isabellina* COR-L2. The respective plant parts and hosts of these strains are leaves of *Sorbus aucuparia*, bark of *Betula pendula*, bark of *Acer platanoides*, bark of *Pinus sylvestris*, and leaves of *Corylus avellana*.

### Identification of the selected endophytic fungi

Five chosen fungal strains with cytotoxic activities, apart from morphological identification, were also identified at the DNA level using the ITS marker. Molecular identification was done using Oxford Nanopore platform with methods thoroughly described in previous paper^19^. After sequencing, revealed ITS sequences were confronted with Mycobank database to identify the species of all samples. Next, a phylogenetic tree was constructed using the identified samples to present their taxonomic position. ITS sequences for other identified species were collected from the NCBI database. Besides, five other species used as outgroups for rooting. Phylogeny was reconstructed using Bayesian approach with MrBayes 3.2.7a software^22^ and the SYM + G substitution model was chosen as the best fit for data by jModelTest^23^. Trees were sampled every 100th MCMC generation for 10,000,000 generations until the average standard deviation of split frequencies was stabilized at a value much below 0.01 for all trees used to construct the consensus tree.

### Effect of ^60^Co gamma irradiation on growth and the cytotoxic potential

As described earlier, spore suspensions of *Penicillium yarmokense* JAR-L1; *Penicillium sp.* BRZ-B; *Fusarium avenaceum* KLON-B, *Penicillium charlesii* SOS-B1; *Umbelopsis isabellina* COR-L2 were prepared then irradiated using a ^60^Co Gamma chamber at dose range 0.5–8 kGy and kept in darkness overnight. All the irradiated suspensions from all doses were separately used and the resultant fungal cultures were extracted and tested as described earlier. Fungal growth was estimated from the resultant fresh biomass after drying to a constant weight (50 °C in a hot air oven). The growth was expressed as g dry biomass L^−1^ culture filtrate.

### Preparative thin-layer chromatography (TLC) fractionation

The fungal extracts with the best cytotoxic potentials (*P. yarmokense* JAR-L1*,* biomass; *Penicillium sp.* sp. BRZ-B, cell-free filtrate; *F. avenaceum* KLON-B, cell-free filtrate; *P. charlesii* SOS-B1, biomass; and *U. isabellina* COR-L2, biomass) were subjected to prep-TLC fractionation (GF-254, Merck, Germany). The fungal extracts were prepared as mentioned earlier then dissolved in 0.5 mL the loaded on the plated and eluted with 9:1, v/v of the mixture chloroform:methanol. The developed TLC plates were examined under longwave (365 nm) and shortwave (245 nm) UV lamps. The separated bands were carefully removed by scraping off the silica gel and eluted with DMSO, then tested again for cytotoxicity against the four cell lines, as described earlier.

### GC–MS analysis

Active fractions from prep-TLC of *P. yarmokense* JAR-L1 (biomass), *Penicillium * sp. BRZ-B (cell-free filtrate), *F. avenaceum* KLON-B (cell-free filtrate), *P. charlesii* SOS-B1 (biomass), and *U. isabellina* COR-L2 (biomass) were analyzed using a Shimadzu GCMS QP 2020 system (Shimadzu, Japan) equipped with a ZB-5 column (30 m × 0.25 mm × 0.25 µm, Phenomenex, Torrance, CA, USA). A 1 µL aliquot of the sample was injected at 260 °C with a split ratio of 100, utilizing helium as the carrier gas at a linear velocity of 35 cm s^−1^. Analyte separation was achieved using a temperature program that started at 50 °C and increased to 250 °C at a rate of 3 °C·min^−1^. The mass spectrometer operated in SCAN mode, covering a mass range of 35–650 m/z. The interface and ion source temperatures were both maintained at 250 °C. Potential analytes were identified by comparing the experimentally obtained mass spectra with those in the NIST20 library (National Institute of Standards and Technology).

### Statistics

Means were calculated from triplicate measurements from two independent experiments and their SD. One-way ANOVA (analysis of variation) followed by least significant difference (LSD) was performed using SPSS V. 22 (IBM, NY) software at the significance level *P* < 0.05.

## Results

### Screening fungal extracts for their cytotoxic potentials

**Fig. 1:**
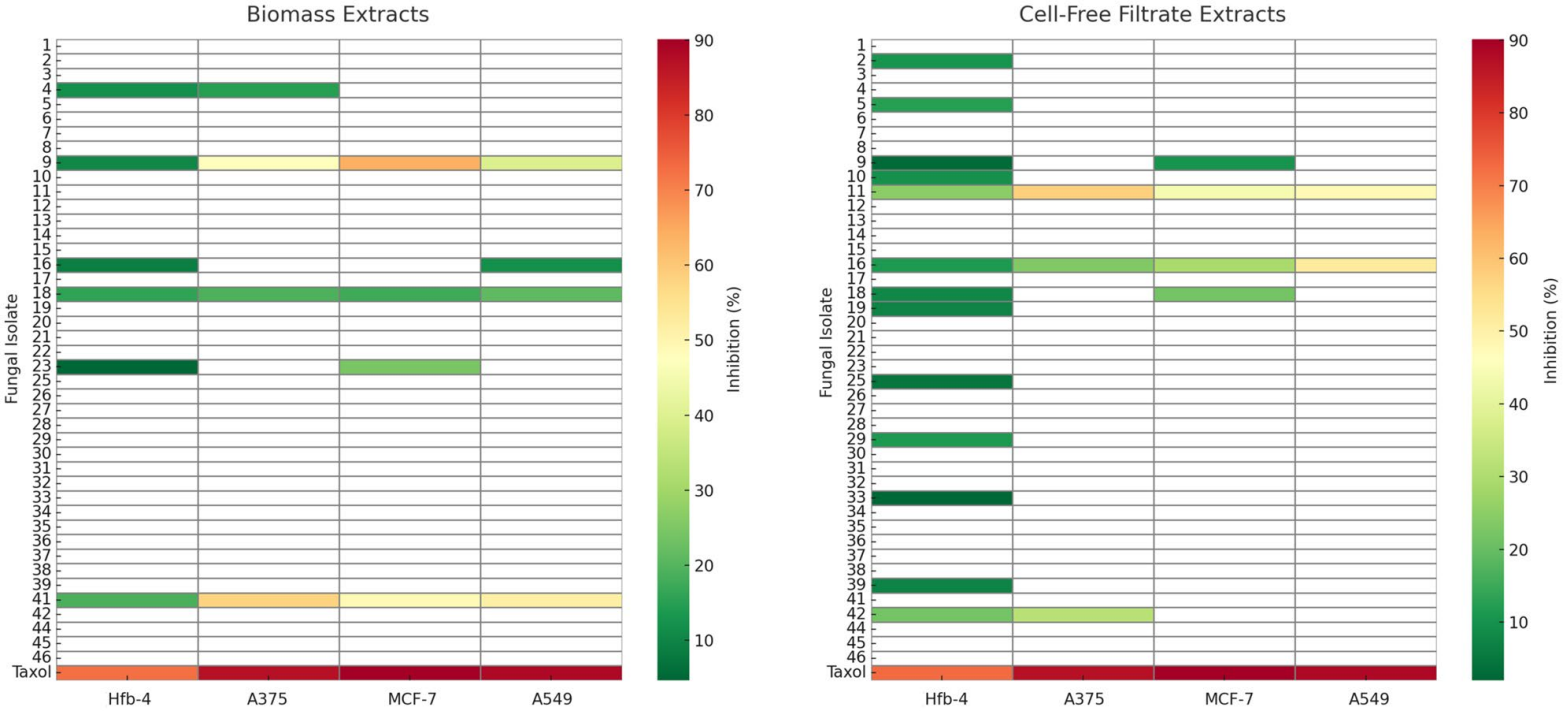
Heat maps illustrating the cytotoxicity data from biomass extracts (left) and cell-free filtrate extracts (right) against normal cell line (Hfb-4), human melanoma (A375), breast adenocarcinoma (MCF-7), and lung carcinoma (A549) cell line.

The cytotoxic activities of the crude fungal extracts (cell-free filtrate and biomass) were evaluated against three different cancer cell lines human melanoma (A375), breast adenocarcinoma (MCF-7), and lung carcinoma (A549) and one normal cell line (Hfb-4) using MTT-based assay. Generally, the obtained results in Tables 1 and 2 and Fig. 1 indicated that cell-free filtrates and biomasses extracts showed variable cytotoxic activities. Data in Table 1 and Fig. 1 show that extracts prepared from the biomass of the fungi number 4, 9, 16, 18, 23 and 41 had potential against all the cell lines where significant differences (*P* < 0.05) were observed. Isolate number 4 showed activity against Hfb-4 (11.98%) and A375 (14.78%) while no activities against MCF-7 and A549. Isolate number 16 showed activity against Hfb-4 (8.76%) and A549 (11.76%) while no activities against MCF-7 and A375. Isolate number 23 showed activity against Hfb-4 (4.67%) and MCF-7 (24.67%) while no activities against A549 and A375. Interestingly, isolates number 9, 18, and 41 showed activity against all cell lines recording 10.56, 15.98, and 18.95% (Hfb-4), 47.51, 19.09, and 57.91% (A375), 63.99, 17.41, and 49.05% (MCF-7), and 39.51, 21.05, and 51.55% (A549), respectively.

**Table 1:** Cytotoxicity of the biomass extracts from the isolated fungal culture against normal cell line (Hfb-4), human melanoma cell line (A375), breast adenocarcinoma cell line (MCF-7), and lung carcinoma cell line (A549).

Fungal isolates	Inhibition (%)			
	Normal (Hfb-4)	Human melanoma (A375)	Breast adenocarcinoma (MCF-7)	Lung carcinoma (A549)
1	Nil	Nil	Nil	Nil
2	Nil	Nil	Nil	Nil
3	Nil	Nil	Nil	Nil
4	11.98 ± 1.45	14.78 ± 1.22	Nil	Nil
5	Nil	Nil	Nil	Nil
6	Nil	Nil	Nil	Nil
7	Nil	Nil	Nil	Nil
8	Nil	Nil	Nil	Nil
9	10.56 ± 0.43	47.51 ± 12.33	63.99 ± 10.81	39.51 ± 9.44
10	Nil	Nil	Nil	Nil
11	Nil	Nil	Nil	Nil
12	Nil	Nil	Nil	Nil
13	Nil	Nil	Nil	Nil
14	Nil	Nil	Nil	Nil
15	Nil	Nil	Nil	Nil
16	8.76 ± 1.08	Nil	Nil	11.76 ± 0.98
17	Nil	Nil	Nil	Nil
18	15.98 ± 1.33	19.09 ± 1.03	17.41 ± 1.63	21.05 ± 2.74
19	Nil	Nil	Nil	Nil
20	Nil	Nil	Nil	Nil
21	Nil	Nil	Nil	Nil
22	Nil	Nil	Nil	Nil
23	4.67 ± 0.21	Nil	24.67 ± 2.85	Nil
25	Nil	Nil	Nil	Nil
26	Nil	Nil	Nil	Nil
27	Nil	Nil	Nil	Nil
28	Nil	Nil	Nil	Nil
29	Nil	Nil	Nil	Nil
30	Nil	Nil	Nil	Nil
31	Nil	Nil	Nil	Nil
32	Nil	Nil	Nil	Nil
33	Nil	Nil	Nil	Nil
35	Nil	Nil	Nil	Nil
36	Nil	Nil	Nil	Nil
37	Nil	Nil	Nil	Nil
38	Nil	Nil	Nil	Nil
39	Nil	Nil	Nil	Nil
41	18.95 ± 5.76	57.91 ± 2.78	49.05 ± 3.76	51.55 ± 2.64
42	Nil	Nil	Nil	Nil
44	Nil	Nil	Nil	Nil
45	Nil	Nil	Nil	Nil
46	Nil	Nil	Nil	Nil
Taxol	72.69 ± 7.33	86.87 ± 1.85	90.15 ± 1.01	88.39 ± 2.07
LSD	4.313	9.891	7.719	9.828

Nil means that no inhibition was detected. Taxol (50 µg mL^−1^) was used as the positive control. The calculated mean is for triplicate measurements ± SD, *LSD* least significant differences (LSD test, *P* ≤ 0.05).

**Table 2:** Cytotoxicity of the cell-free filtrate extracts from the isolated fungal culture against normal cell line (Hfb-4), human melanoma cell line (A375), breast adenocarcinoma cell line (MCF-7), and lung carcinoma cell line (A549).

Fungal isolates	Inhibition (%)			
	Normal (Hfb-4)	Human melanoma (A375)	Breast adenocarcinoma (MCF-7)	Lung carcinoma (A549)
1	Nil	Nil	Nil	Nil
2	10.32 ± 1.44	Nil	Nil	Nil
3	Nil	Nil	Nil	Nil
4	Nil	Nil	Nil	Nil
5	12.51 ± 0.93	Nil	Nil	Nil
6	Nil	Nil	Nil	Nil
7	Nil	Nil	Nil	Nil
8	Nil	Nil	Nil	Nil
9	2.97 ± 0.11	Nil	10.44 ± 2.51	Nil
10	9.67 ± 1.58	Nil	Nil	Nil
11	24.77 ± 9.57	57.56 ± 10.89	43.71 ± 8.91	47.67 ± 11.67
12	Nil	Nil	Nil	Nil
13	Nil	Nil	Nil	Nil
14	Nil	Nil	Nil	Nil
15	Nil	Nil	Nil	Nil
16	11.57 ± 1.09	23.44 ± 8.65	29.55 ± 4.76	51.78 ± 10.03
17	Nil	Nil	Nil	Nil
18	7.98 ± 1.78	Nil	21.78 ± 4.89	Nil
19	7.45 ± 0.33	Nil	Nil	Nil
20	Nil	Nil	Nil	Nil
21	Nil	Nil	Nil	Nil
22	Nil	Nil	Nil	Nil
23	Nil	Nil	Nil	Nil
25	4.99 ± 0.39	Nil	Nil	Nil
26	Nil	Nil	Nil	Nil
27	Nil	Nil	Nil	Nil
28	Nil	Nil	Nil	Nil
29	11.59 ± 1.65	Nil	Nil	Nil
30	Nil	Nil	Nil	Nil
31	Nil	Nil	Nil	Nil
32	Nil	Nil	Nil	Nil
33	2.01 ± 0.08	Nil	Nil	Nil
35	Nil	Nil	Nil	Nil
36	Nil	Nil	Nil	Nil
37	Nil	Nil	Nil	Nil
38	Nil	Nil	Nil	Nil
39	7.21 ± 0.19	Nil	Nil	Nil
41	Nil	Nil	Nil	Nil
42	21.66 ± 4.67	31.89 ± 2.33	Nil	Nil
44	Nil	Nil	Nil	Nil
45	Nil	Nil	Nil	Nil
46	Nil	Nil	Nil	Nil
Taxol	72.69 ± 7.33	86.87 ± 1.85	90.15 ± 1.01	88.39 ± 2.07
LSD	4.313	9.891	7.719	9.828

Nil means that no inhibition was detected. Taxol (50 µg mL^−1^) was used as the positive control. The calculated mean is for triplicate measurements ± SD, *LSD* least significant differences (LSD test, *P* ≤ 0.05).

Regarding the cell-free filtrate, results presented in Table 2 and Fig. 1 indicate that the fungi numbers 2, 5, 9, 11, 16, 18, 19, 25, 29, 33, 39 and 42 had potentials against Hfb-4 recording 10.32, 12.51, 2.97, 9.67, 24.77, 11.57, 7.98, 7.45, 4.99, 11.59, 2.01, 7.21, and 21.66%, respectively; where significant differences (*P* < 0.05) were observed. Interestingly, isolates number 11 and 16 showed activity against Hfb-4 (24.77 and 11.57%), A375 (57.56 and 23.44%), MCF-7 (43.71 and 29.55%), and A549 (47.67 and 51.78%), respectively. Accordingly, the biomass-based extracts of fungi 9, 18, and 41 and the cell free filtrate-based extracts of fungi 11 and 16 were chosen for subsequent analysis.

### Identification of the selected fungal strains

**Fig. 2:**
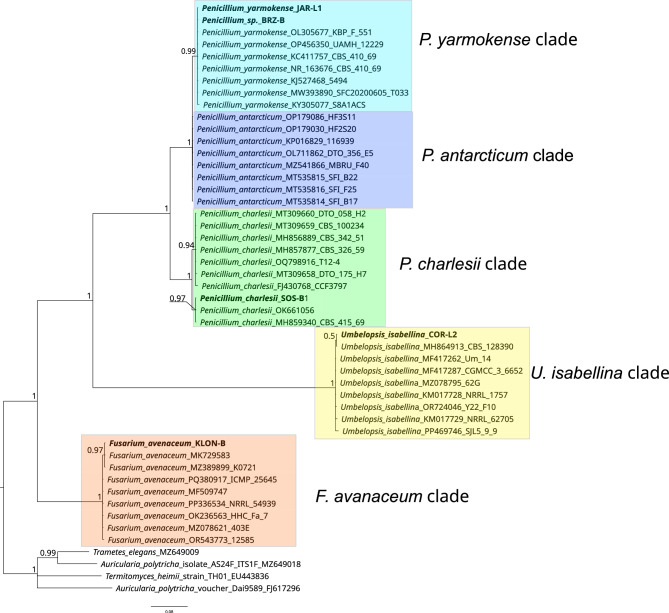
Bayesian phylogenetic tree of the ITS sequences of the JAR-L1, BRZ-B, KLON-B, SOS-B1, and COR-L2 isolates and sequences from NCBI. Posterior probabilities of the nodes are shown along the nodes. Different colors are showing distinct clades and samples analyzed in this study are bolded.

Based on their promising activities, selected fungal strains 9, 11, 16, 18, and 41 were identified using molecular identification. After sequencing, concatenated ITS sequences for each identified strain were created. Samples of the identified fungi were deposited under accession numbers PV163118, PV163119, PV163120, PV163121, and PV163122 in the NCBI database. Fig. 2   shows that four out of five analyzed strains grouped with other representatives of the same genera on the tree had high probability. Those strains are *Penicillium yarmokense* JAR-L1, Fusarium* avenaceum* KLON-B, *Penicillium charlesii* SOS-B1, and *Umbelopsis isabellina* COR-L2. One strain—BRZ-B, was not confirmed. where it showed 100% identity with several different *Penicillium* species and did not group with *Penicillium antarcticum* clade on the phylogenetic tree. Thus, we described the species as *Penicillium* sp. (Fig. 2).

### Gamma irradiation effect on growth and the cytotoxic potential

Several doses (0.5–8 KGy) of gamma irradiation were used to study their effect on fungal growth and the cytotoxic potentials of extracts *P. yarmokense* JAR-L1 (biomass), *F. avenaceum* KLON-B (cell-free filtrate),* Penicillium * sp. BRZ-B (cell-free filtrate), *P. charlesii* SOS-B1 (biomass), and *U. isabellina* COR-L2 (biomass) against normal cell line (Hfb-4), human melanoma cell line (A375), breast adenocarcinoma cell line (MCF-7), and lung carcinoma cell line (A549). Notably, the remarkable feature of the results in Table 3 is the enhancing effect of gamma irradiation on the cytotoxic potential against the four cell lines of extracts from the five fungi. Nevertheless, the irradiation dose that results in maximum inhibition values depends on the fungus. For example, 1000 Gy was the best dose for maximum inhibition by *P. yarmokense* JAR-L1 and *F. avenaceum* KLON-B against normal cell line (21.78 and 16.88%), human melanoma cell line (76.89 and 37.98%), breast adenocarcinoma cell line (75.34 and 47.88%), and lung carcinoma cell line (58.09 and 69.32%). Regarding *Penicillium * sp. BRZ-B, *P. charlesii* SOS-B1, and *U. isabellina* COR-L2, 2000 Gy was the best dose for maximum inhibition against normal cell line (32.32, 17.65, and 68.88%), human melanoma cell line (75.43, 87.65, and 69.97%), breast adenocarcinoma cell line (65.98, 71.06, and 84.92%), and lung carcinoma cell line (77.98, 76.39, and 83.86%). Moreover, no activities were recorded at the higher doses 4000 by *P. yarmokense* JAR-L1, *F. avenaceum* KLON-B, and *U. isabellina* COR-L2 and at 8000 Gy by *Penicillium * sp. BRZ-B, *P. charlesii* SOS-B1 (Table 3).

**Table 3:** Effect of gamma irradiation on growth (g L^−1^) and the cytotoxic potentials of extracts *P. yarmokense* JAR-L1 (biomass), *Penicillium * sp. BRZ-B (cell-free filtrate), *F. avenaceum* KLON-B (cell-free filtrate), *P. charlesii* SOS-B1 (biomass), and *U. isabellina* COR-L2 (biomass) against normal cell line (Hfb-4), human melanoma cell line (A375), breast adenocarcinoma cell line (MCF-7), and lung carcinoma cell line (A549).

Fungal strain	Dose (kGy)	Dry biomass (g L^−1^)	Inhibition (%)			
			Normal (Hfb-4)	Melanoma (A375)	Breast (MCF-7)	Lung (A549)
*P. yarmokense* JAR-L1 (biomass)	0.0 (C)	13.78 ± 1.78	10.42 ± 0.88	47.65 ± 10.87	62.76 ± 12.44	38.98 ± 7.45
	0.5	13.89 ± 1.45	10.87 ± 2.23	48.98 ± 12.78	62.89 ± 11.54	42.66 ± 15.32
	1	9.78 ± 0.66	21.78 ± 0.67	76.89 ± 15.32	75.34 ± 10.78	58.09 ± 12.81
	2	2.67 ± 0.06	2.55 ± 0.32	5.89 ± 0.71	11.89 ± 2.34	4.55 ± 0.78
	4	1.55 ± 0.08	Nil	Nil	Nil	Nil
	8	0.00	Nil	Nil	Nil	Nil
*Penicillium sp.* BRZ-B (cell-free filtrate)	0.0 (C)	14.76 ± 2.67	20.63 ± 10.43	55.77 ± 12.65	45.08 ± 12.89	46.88 ± 10.66
	0.5	15.34 ± 1.78	22.89 ± 9.78	56.32 ± 13.98	46.33 ± 12.43	54.90 ± 10.43
	1	10.56 ± 0.67	23.78 ± 7.87	68.78 ± 16.11	58.98 ± 15.08	59.13 ± 18.78
	2	8.78 ± 0.45	32.32 ± 0.11	75.43 ± 14.78	65.98 ± 14.78	77.98 ± 15.67
	4	1.88 ± 0.07	1.01 ± 0.05	10.56 ± 2.98	9.98 ± 0.75	2.99 ± 0.95
	8	0.00	Nil	Nil	Nil	Nil
*F. avenaceum* KLON-B (cell-free filtrate)	0.0 (C)	12.67 ± 1.59	10.90 ± 1.99	24.09 ± 2.77	28.98 ± 1.51	50.01 ± 5.89
	0.5	10.07 ± 0.61	10.89 ± 3.89	28.98 ± 11.98	28.78 ± 8.36	51.98 ± 10.87
	1	8.89 ± 0.86	16.88 ± 3.87	37.98 ± 12.65	47.88 ± 10.98	69.32 ± 13.65
	2	4.97 ± 0.74	5.99 ± 1.52	10.98 ± 2.89	3.98 ± 0.61	10.87 ± 3.44
	4	1.09 ± 0.04	Nil	Nil	Nil	Nil
	8	0.00	Nil	Nil	Nil	Nil
*P. charlesii* SOS-B1 (biomass)	0.0 (C)	13.89 ± 1.55	13.44 ± 2.67	18.22 ± 1.76	17.27 ± 2.88	21.18 ± 3.95
	0.5	13.67 ± 2.76	14.87 ± 3.87	19.87 ± 2.34	18.89 ± 3.07	43.57 ± 10.87
	1	10.87 ± 1.44	12.88 ± 3.55	32.88 ± 10.76	29.34 ± 10.77	54.98 ± 13.78
	2	6.67 ± 0.09	17.65 ± 2.98	87.65 ± 12.11	71.06 ± 14.76	76.39 ± 16.78
	4	1.12 ± 0.11	4.99 ± 0.45	44.99 ± 10.63	37.67 ± 10.65	42.61 ± 6.98
	8	0.00	Nil	Nil	Nil	Nil
*U. isabellina* COR-L2 (biomass)	0.0 (C)	10.55 ± 1.43	15.77 ± 3.78	58.32 ± 11.32	47.59 ± 7.32	50.92 ± 14.88
	0.5	9.89 ± 0.57	38.66 ± 10.65	59.98 ± 15.76	54.99 ± 10.76	52.87 ± 13.97
	1	8.43 ± 0.39	56.89 ± 11.59	66.4 ± 14.93	61.97 ± 11.93	63.98 ± 14.31
	2	6.98 ± 1.68	68.88 ± 14.89	69.97 ± 13.81	84.92 ± 13.21	83.86 ± 12.86
	4	2.01 ± 0.43	Nil	Nil	Nil	Nil
	8	0.00	Nil	Nil	Nil	Nil
Taxol			74.98 ± 15.66	86.49 ± 11.47	90.21 ± 10.17	89.01 ± 12.65
LSD		0.321	6.081	10.948	10.739	11.883

Nil means that no inhibition was detected. Taxol (50 µg mL^−1^) was used as the positive control. The calculated mean is for triplicate measurements ± SD, *LSD* Least Significant Differences (LSD test, *P* ≤ 0.05).

Regarding the effect of gamma irradiation on the fungal growth, our results indicated the dose-related manner. Moreover, the survival rates of *P. yarmokense* JAR-L1, *F. avenaceum* KLON-B, *Penicillium * sp. BRZ-B, *P. charlesii* SOS-B1, and *U. isabellina* COR-L2 were similarly responded to the applied doses of gamma rays. At 0.5 kGy, there were no significant differences from the non-irradiated cultures. At 4 kGy, the recorded fungal growth was significantly reduced. Meanwhile, no growth was recorded at 8 kGy (Table 3).

### Chemical analysis of active fractions

Extracts of the biomass of *P. yarmokense* JAR-L1, cell-free filtrate of *Penicillium * sp. BRZ-B, cell-free filtrate of *F. avenaceum* KLON-B, biomass of *P. charlesii* SOS-B1, and biomass of *U. isabellina* COR-L2 were subjected to prep-TLC and the collected fractions were tested again for cytotoxic potentials to detect the active bands. Table 4 shows that active bands for the respective fungi were bands number 3, 3, 1, 2, and 2. The recorded activities of the respective bands against Hfb-4 were 10.21, 22.89, 10.89, 7.45, and 17.56%, against A375 were 42.67, 55.08, 16.98, 10.21, and 50.34%, against MCF-7 were 53.77, 41.89, 25.78, 10.67, and 43.21%, and against A549 were 37.99, 40.39, 38.08, 1.89, and 48.67%.

**Table 4:** Cytotoxicity of prep-TLC fractions from extracts of *P. yarmokense* JAR-L1 (biomass), *Penicillium * sp. BRZ-B (cell-free filtrate), *F. avenaceum* KLON-B (cell-free filtrate), *P. charlesii* SOS-B1 (biomass), and *U. isabellina* COR-L2 (biomass) against normal cell line (Hfb-4), human melanoma cell line (A375), breast adenocarcinoma cell line (MCF-7), and lung carcinoma cell line (A549).

Fungal strain	TLC Fraction No	Inhibition (%)			
		Normal (Hfb-4)	Melanoma (A375)	Breast (MCF-7)	Lung (A549)
*P. yarmokense* JAR-L1 (biomass)	1	Nil	Nil	Nil	Nil
	2	Nil	Nil	Nil	Nil
	3	10.21 ± 1.67	42.67 ± 10.31	53.77 ± 6.29	37.99 ± 3.85
*Penicillium sp.* BRZ-B (cell-free filtrate)	1	Nil	Nil	Nil	Nil
	2	Nil	Nil	Nil	Nil
	3	22.89 ± 3.21	55.08 ± 11.43	41.89 ± 9.45	40.39 ± 10.51
	4	Nil	Nil	Nil	Nil
*F. avenaceum* KLON-B (cell-free filtrate)	1	10.89 ± 2.11	16.98 ± 2.43	25.78 ± 11.33	38.08 ± 14.21
	2	Nil	Nil	Nil	Nil
	3	Nil	Nil	Nil	Nil
	4	Nil	Nil	Nil	Nil
	5	Nil	Nil	Nil	Nil
*P. charlesii* SOS-B1 (biomass)	1	Nil	Nil	Nil	Nil
	2	7.45 ± 0.78	10.21 ± 0.89	10.67 ± 1.71	1.89 ± 0.55
	3	Nil	Nil	Nil	Nil
	4	Nil	Nil	Nil	Nil
	5	Nil	Nil	Nil	Nil
	6	Nil	Nil	Nil	Nil
	7	Nil	Nil	Nil	Nil
	8	Nil	Nil	Nil	Nil
*U. isabellina* COR-L2 (biomass)	1	Nil	Nil	Nil	Nil
	2	17.56 ± 2.97	50.34 ± 7.56	43.21 ± 7.89	48.67 ± 8.55
	3	Nil	Nil	Nil	Nil
Taxol		72.58 ± 14.99	86.07 ± 12.79	91.01 ± 12.66	88.58 ± 13.41
LSD		2.519	10.662	8.398	9.985

Nil means that no inhibition was detected. Taxol (50 µg mL^−1^) was used as the positive control. The calculated mean is for triplicate measurements ± SD, *LSD* least significant differences (LSD test, *P* ≤ 0.05).

**Fig. 3:**
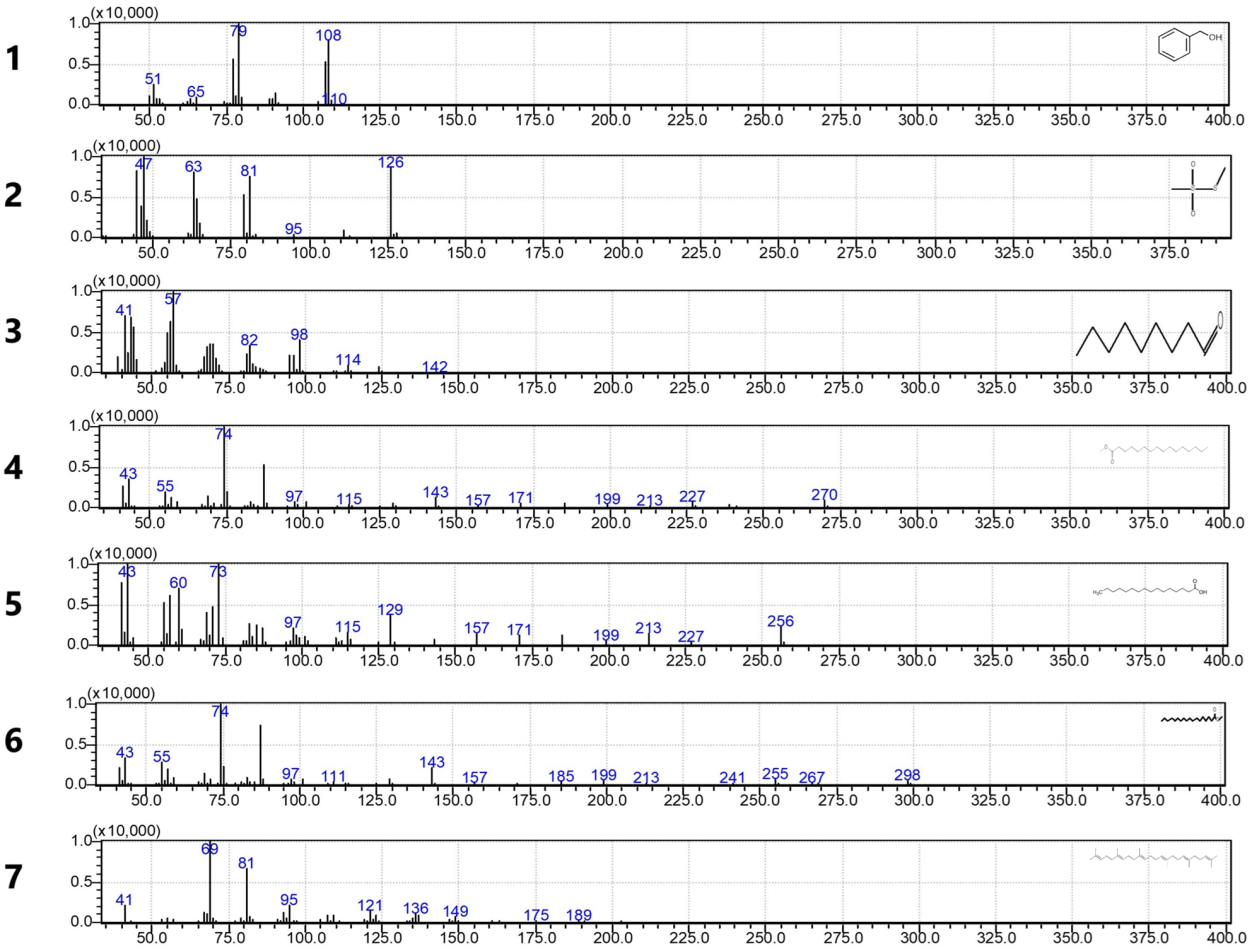
GC–MS spectra of the detected compounds from active band of the *P. yarmokense* JAR-L1 biomass extract. Benzyl alcohol (**1**), S-Methyl methanethiosulphonate (**2**), Nonanal (**3**), Hexadecanoic acid, methyl ester (**4**), Hexadecanoic acid (**5**), Octadecanoic acid, methyl ester (**6**), and Squalene (**7**).

**Fig. 4:**
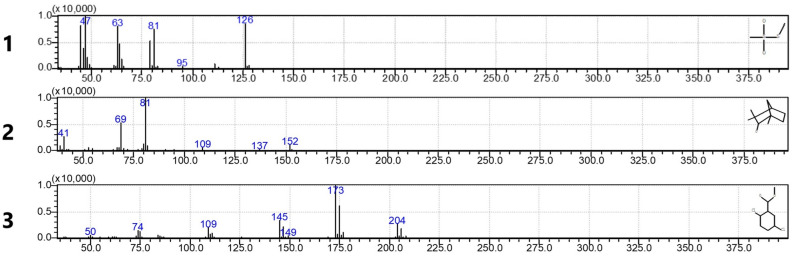
GC–MS spectra of the detected compounds from active band of the *Penicillium * sp. BRZ-B cell-free filtrate extract. S-Methyl methanethiosulphonate (**1**), Fenchone (**2**), and Methyl 2,5-dichlorobenzoate (**3**).

**Fig. 5:**
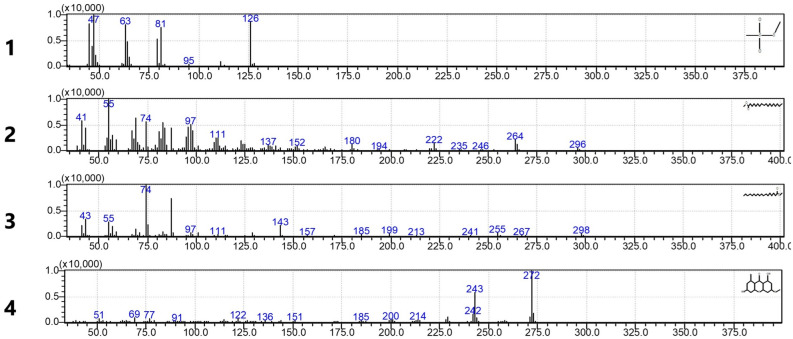
GC–MS spectra of the detected compounds from active band of the *F. avenaceum* KLON-B cell-free filtrate extract. α-Phellandrene (**1**), S-Methyl methanethiosulphonate (**2**), Hexadecanoic acid, methyl ester (**3**), and 9,12-Octadecadienoic acid (Z,Z)-, methyl ester (**4**).

**Fig. 6:**
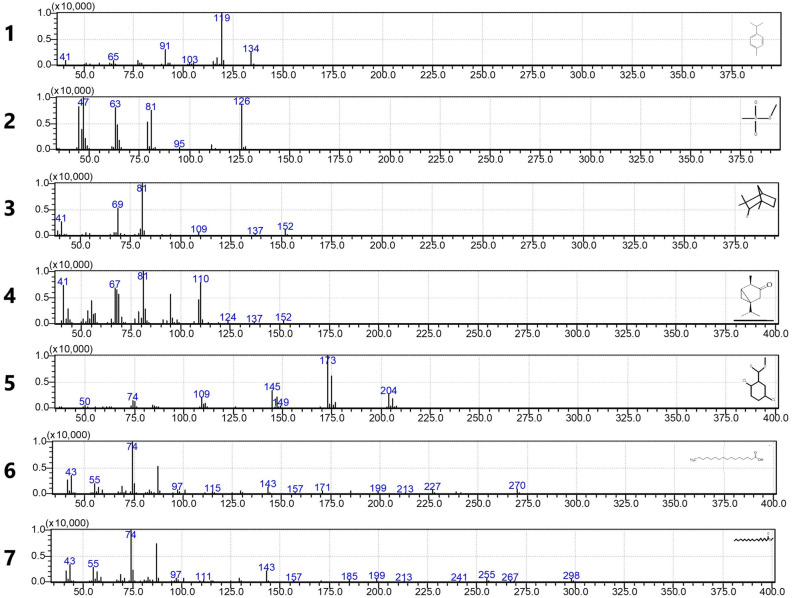
GC–MS spectra of the detected compounds from active band of the *P. charlesii* SOS-B1 biomass extract. *p*-Cymene (**1**), S-Methyl methanethiosulphonate (**2**), Fenchone (**3**), α-Thujone (**4**), Benzoic acid, 2,5-dichloro-, methyl ester (**5**), Hexadecanoic acid, methyl ester (**6**), and Octadecanoic acid, methyl ester (**7**).

**Fig. 7:**
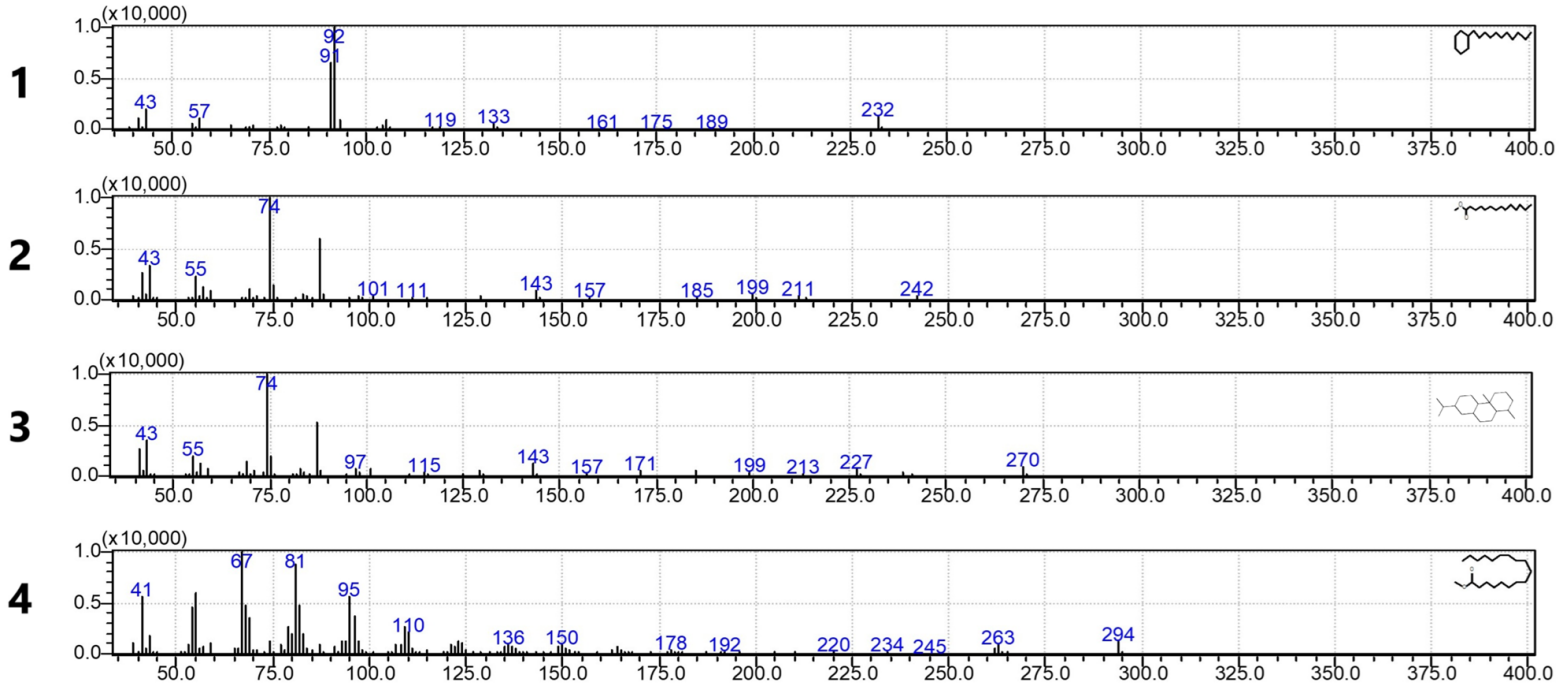
GC–MS spectra of the detected compounds from active band of the *U. isabellina* COR-L2 biomass extract. Benzene, undecyl- (**1**), Hexadecanoic acid, methyl ester (**2**), 18-Norabietane (**3**), and 9,12-Octadecadienoic acid (Z,Z)-, methyl ester (**4**).

These bands from each fungal strain were analyzed by GC–MS. Table 5 shows the detected compounds from each fungus and their retention times. Moreover, Figs. 3, 4, 5, 6 and 7 show GC–MS spectra of *P. yarmokense* JAR-L1 (Fig. 3), *Penicillium * sp. BRZ-B (Fig. 4), *F. avenaceum* KLON-B (Fig. 5), *P. charlesii* SOS-B1 (Fig. 6), and *U. isabellina* COR-L2 (Fig. 7). The results revealed the existence of a wide variety of well-known compounds such as squalene (from *P. yarmokense* JAR-L1), fenchone (from *Penicillium * sp. BRZ-B), α-phellandrene (from *F. avenaceum* KLON-B), *p*-Cymene, fenchone, and α-thujone (from *P. charlesii* SOS-B1), and 18-norabietane (from *U. isabellina* COR-L2).

**Table 5:** GC–MS analysis of active bands of prep-TLC fractions from *P. yarmokense* JAR-L1, *Penicillium * sp. BRZ-B, *F. avenaceum* KLON-B, *P. charlesii* SOS-B1, and *U. isabellina* COR-L2.

Fungal strain	Extract type	S.N	RT (min)	Detected compounds
*P. yarmokense* JAR-L1	Biomass	1	13.69	Benzyl alcohol
		2	14.5	*S*-methyl methanethiosulfonate
		3	15.6	Nonanal
		4	48.5	Hexadecanoic acid, methyl ester
		5	49.6	Hexadecanoic acid
		6	54.75	Octadecanoic acid, methyl ester
		7	63.3	Squalene
*Penicillium * sp. BRZ-B	Cell-free filtrate	1	14.5	*S*-Methyl methanethiosulfonate
		2	15	Fenchone
		3	29	Methyl 2,5-dichlorobenzoate
*F. avenaceum* KLON-B	Cell-free filtrate	1	11.3	α-Phellandrene
		2	14.5	*S*-Methyl methanethiosulfonate
		3	48.5	Hexadecanoic acid, methyl ester
		4	53.68	9,12-Octadecadienoic acid (*Z*,*Z*)-, methyl ester
*P. charlesii* SOS-B1	Biomass	1	12.1	*p*-Cymene
		2	14.5	*S*-Methyl methanethiosulfonate
		3	15	Fenchone
		4	15.8	α-Thujone
		5	28.9	Benzoic acid, 2,5-dichloro-, methyl ester
		6	48.5	Hexadecanoic acid, methyl ester
		7	54.75	Octadecanoic acid, methyl ester
*U. isabellina* COR-L2	Biomass	1	43.4	Benzene, undecyl-
		2	48.5	Hexadecanoic acid, methyl ester
		3	48.6	18-Norabietane
		4	53.68	9,12-Octadecadienoic acid (*Z*,*Z*)-, methyl ester

## Discussion

In the current scenario, the search for natural anticancer compounds is of paramount importance due to their potential to offer effective, selective, and less toxic alternatives to conventional chemotherapy^5^. Many natural products, derived from plants, marine organisms, and microorganisms, have historically served as the foundation for anticancer drug development^6^. Additionally, the vast biodiversity of endophytic fungi of forest plants provides an untapped reservoir of novel bioactive molecules that could overcome drug resistance, a major challenge in cancer therapy. As the global cancer burden continues to rise, investing in the discovery and development of natural anticancer compounds remains a crucial avenue for improving patient outcomes and advancing precision medicine. With this view, extracts (cell-free filtrate and biomass) from 43 endophytic fungi cultures were checked for their cytotoxic activities against three different cancer cell lines and one normal cell line using MTT-based assay. Our results indicated that cell-free filtrates and biomasses extracts showed variable cytotoxic activities where several fungi showed different potentials against the tested cell lines. Our data further showed that only five fungal extracts namely 9, 11, 16, 18, and 41 showed selective cytotoxic activities against the tested cell lines. In the literature, endophytic fungi have garnered significant attention, with their isolation, cultivation, purification, and characterization uncovering approximately 200 diverse and important metabolites, including anticancer agents^24^. Research has identified several anticancer compounds derived from endophytic fungi including taxol^25,26^, camptothecin^27^, vinblastine^28^, podophyllotoxin^29^, and fusarubin^30^ owing to the fascinating biodiversity of such endophytes. Many of these anticancer compounds are currently applied in cancer treatment including breast, lung, prostate, ovarian cancers, and leukemia^31^.

Here, the five fungi with the promising activities were identified as *Penicillium yarmokense* JAR-L1, *Penicillium * sp. BRZ-B, *Fusarium avenaceum* KLON-B, *Penicillium charlesii* SOS-B1, and *Umbelopsis isabellina* COR-L2. In the literature, the genera of fungal endophytes containing putative producers of anticancer agents are* Alternaria*, *Aspergillus*, *Chaetomium*, *Ceriporia*, *Colletotrichum*, *Acremonium*, *Emericella*, *Cytospora*, *Eutypella*, *Epicoccum*^26^, *Eurotium*, *Fusarium*, *Guignardia*, *Hypocrea*, *Penicillium*, *Pestalotiopsis*, *Phomopsis*^24^, *Stemphylium*, *Periconia*,* Thielavia*, and* Talaromyces*, and *Xylaria*^31^.

In the effort to enhance the recorded activities, several doses (0.5–8 KGy) of gamma irradiation were used to study their effect on fungal growth and the cytotoxic potentials of extracts *P. yarmokense* JAR-L1 (biomass), *F. avenaceum* KLON-B (cell-free filtrate),* Penicillium * sp. BRZ-B (cell-free filtrate), *P. charlesii* SOS-B1 (biomass), and *U. isabellina* COR-L2 (biomass) against normal cell line (Hfb-4), human melanoma cell line (A375), breast adenocarcinoma cell line (MCF-7), and lung carcinoma cell line (A549). Our results demonstrated the enhancing effect of gamma irradiation on the cytotoxic potential against the four cell lines. Moreover, the best dose for maximum inhibition values depends on the tested fungus. In agreement with our results, 1 and 2 KGy gamma rays were used to significantly enhance the antioxidant and antimicrobial potentials of extracts from the endophytes *Trichoderma harzianum*, *Aspergillus ochraceus*, *Chaetomium cochliodes*, *Fusarium tricinctum*, and *Penicillium chrysogenum*^19^. Generally, gamma radiation is a highly potent form of ionizing energy^32,33^. When exposing living cells to this radiation (at specific doses), mutagenesis occurs through several mechanisms such as DNA repair^34,35^. Recently, gamma-ray-induced random mutagenesis was used for enhancing production capabilities of several microbial strains^36–38^. Random mutagenesis involves an iterative exposure to chemical or physical mutagens, yielding a phenotypic and genetic diversity of daughters, which have to be screened for the improved metabolic functions^39^. Our findings highlight that exposing endophytic fungi to gamma irradiation (1–2 kGy) can stimulate the production of bioactive compounds, likely by inducing mutations in biosynthetic gene clusters^34,37^. Notably, the enhancement of cytotoxic activity in *Penicillium yarmokense* and *Fusarium avenaceum* could be correlated with an increased abundance of squalene and α-phellandrene, respectively. These metabolites have documented anticancer properties, primarily by modulating oxidative stress and apoptosis pathways^40,41^. Although higher doses of radiation (≥ 4 kGy) were detrimental to fungal growth and secondary metabolism, this study underscores the potential of moderate gamma irradiation in strain improvement for drug discovery.

In the current study, extracts of the biomass of *P. yarmokense* JAR-L1, cell-free filtrate of *Penicillium * sp. BRZ-B, cell-free filtrate of *F. avenaceum* KLON-B, biomass of *P. charlesii* SOS-B1, and biomass of *U. isabellina* COR-L2 were subjected to prep-TLC and the collected fractions were tested again for cytotoxic potentials to detect the active bands then analyzed by GC–MS. Our results confirmed the existence of a wide variety of compounds. Previous reports listed a plethora of anti-cancer compounds from fungal endophytes such as alkaloids, terpenes, lignans^20^, polyketides, quinones, polyphenols, tetralones, peptides, etc.^27^. Importantly, our results confirmed the existence of squalene, fenchone, α-phellandrene, *p*-Cymene, α-thujone, and 18-norabietane in extracts from *P. yarmokense* JAR-L1, *Penicillium sp.* BRZ-B, *F. avenaceum* KLON-B, *P. charlesii* SOS-B1 and *U. isabellina* COR-L2. To the best of our knowledge, there is no data on these compounds from the mentioned fungal species. Squalene, an isoprenoid compound structurally similar to β-carotene, is an intermediate metabolite in the synthesis of cholesterol^40^. The primary therapeutic use of squalene currently is as an adjunctive therapy in a variety of cancers^42^. Fenchone, a bicyclic monoterpene ketone has anti-inflammatory^43^, antioxidant^44^, antinociceptive potentials^45^. The biological functions of α-phellandrene showed antimicrobial, anti-inflammatory, wound healing, and anticancer potentials^41^. *p*-Cymene has antioxidant, anti-inflammatory, antimicrobial, anti-tumor^46^, α-thujone has anti-tumor activities^47^, and 18-norabietane is a diterpene perhydrogenated phenanthrene derivative and one of the abietanes which have a wide variety of interesting biological activities^48^. Our findings need more confirmation of the detected compounds by other analytical techniques such as NMR and LC–MS analysis.

## Conclusions

Five different endophytic fungi showed bioactive metabolites with cytotoxic potentials against breast adenocarcinoma, human melanoma, lung carcinoma, and normal cell line. The cytotoxic potentials of these fungi were intensified after exposure to gamma rays at specific doses. Preparative TLC followed by GC–MS was used to identify bioactive compounds. Our results strongly recommend bioprospecting forest plants associated fungal endophytes to discovering untapped sources of anticancer compounds. Moreover, the presented research highlights the great potential of such unique environments, an exploration of which can lead to discovering new anticancers and broadening scientific knowledge. Current works is in progress on upscaling fungal fermentation, further purification of potent fractions, confirmation by NMR and LC–MS analyses and in vivo studies to assess therapeutic efficacy and toxicity profiles.

## Data Availability

The authors declare that the data supporting the findings of this study are available within the paper.
